# Circulating metabolite biomarkers associated with prostate cancer risk in Black Americans: findings from the Southern Community Cohort Study

**DOI:** 10.1038/s41416-026-03520-z

**Published:** 2026-06-27

**Authors:** Hyung-Suk Yoon, Xiao-Ou Shu, Jie Wu, Maureen Sanderson, Xiaofei Wang, Wanqing Wen, Regina Courtney, Jae Jeong Yang, William J. Blot, Wei Zheng, Qiuyin Cai

**Affiliations:** 1 Department of Surgery, College of Medicine, University of Florida, Gainesville, FL USA; 2 University of Florida Health Cancer Institute, Gainesville, FL USA; 3 Division of Epidemiology, Department of Medicine, Vanderbilt Epidemiology Center, Vanderbilt-Ingram Cancer Center, Vanderbilt University School of Medicine, Nashville, TN USA; 4 Department of Family & Community Medicine, Meharry Medical College, Nashville, TN USA; 5 Department of Biological Sciences, Tennessee State University, Nashville, TN USA

**Keywords:** Risk factors, Predictive markers

## Abstract

**Background:**

Despite the importance of metabolic reprogramming in prostate cancer initiation and progression, little is known about metabolic features associated with prostate cancer risk among Black Americans.

**Methods:**

This nested case-control study included 195 incident prostate cancer cases and 379 matched controls, focusing on Black Americans from the Southern Community Cohort Study. Using pre-diagnostic plasma samples, a global, semi-quantitative metabolomics assay was conducted. Multivariable-adjusted conditional logistic regression was used to estimate odds ratios (ORs) and 95% confidence intervals (95% CIs) for prostate cancer risk associated with a one standard-deviation increase in log-transformed metabolite levels.

**Results:**

A total of 26 metabolites were associated with incident prostate cancer at *p* < 0.01. Of them, seven metabolites were independently associated with prostate cancer risk after mutual adjustment, including S-adenosylhomocysteine (SAH; OR [95% CI] = 0.69 [0.55–0.88]), 14-HDoHE/17-HDoHE (1.46 [1.17–1.83]), 1-linoleoyl-GPI (18:2)* (0.73 [0.57–0.92]), Sphingomyelin (d17:1/16:0, d18:1/15:0, d16:1/17:0)* (1.28 [1.03–1.59]), Gamma-glutamyl-epsilon-lysine (1.45 [1.14–1.85]), and 2,2’-Methylenebis(6-tert-butyl-p-cresol) (0.62 [0.40–0.95]). SAH and 14-HDoHE/17-HDoHE were exclusively linked to aggressive prostate cancer, while the other five metabolites were primarily associated with non-aggressive forms.

**Conclusions:**

We found several circulating metabolites as potential biomarkers for prostate cancer risk among Black Americans. Further large-scale studies are warranted to confirm our findings and to elucidate potential mechanisms.

## Background

Prostate cancer is the most common malignancy and the second leading cause of cancer-related death among men in the US [1]. In 2025, about 313,780 new diagnoses of prostate cancer are projected, representing 30% of all new cancers developed among US males, with an estimated 35,770 deaths from the disease [1, 2]. Of note, Black American males carry a disproportionate burden of prostate cancer, facing a 2.2-fold increased risk of dying due to this cancer compared to their White counterparts [3–5]. They also possess a significantly higher likelihood of being diagnosed at a younger age [6] and are often predisposed to aggressive, fast-growing tumor subtypes [7, 8]. Prostate cancer predominantly affects Black Americans more than any other racial or ethnic group in the US; nevertheless, the underlying mechanisms driving these persistent disparities remain poorly understood.

An emerging body of evidence highlights metabolic reprogramming in the development and progression of prostate cancer [9–13]. Previous research has discovered multiple blood and tissue metabolites associated with prostate cancer risk, embracing a range of signaling pathways, such as cellular growth and proliferation, energy metabolism, amino acid metabolism, and steroid hormone regulation [14–20]; however, these studies have predominantly focused on males of European descent, with minimal representation of Black Americans. A few studies on Black Americans have reported specific metabolites in estrogen, vitamin D, and lipid metabolism that are potentially relevant to prostate cancer [21–23], although limited by small sample sizes, a narrow array of selected metabolites, and cross-sectional designs. Currently, little is known about metabolic characteristics associated with prostate cancer risk among Black Americans. A comprehensive metabolomics analysis targeting Black American males is critical to elucidate perhaps distinct metabolites linked to their elevated risk of prostate cancer. The primary objective of this nested case-control study on untargeted blood metabolomics is to explore circulating metabolite biomarkers for prostate carcinogenesis among Black American males, a population disproportionately affected by the disease.

## Methods

### Study population

This study is based on the Southern Community Cohort Study (SCCS), a prospective, population-based cohort to investigate the fundamental causes of persistent health disparities across Black Americans and low-income individuals. Detailed information on the SCCS has been reported in previous articles [24, 25]. After providing written informed consent, men and women residing in 12 southeastern states of the US were enrolled (*n* ~ 85,000). Approximately 86% of the participants were recruited from community health centers (CHCs) that offer primary healthcare services for predominantly low-income and uninsured populations. The remaining 14% were enrolled via mailings from a random sample of the general population in the same regions. Baseline information on sociodemographic characteristics, anthropometrics, medical history, and health behaviors (e.g., smoking, alcohol consumption, physical activity, and dietary habits) was collected through computer-assisted personal interviews. Almost half of the participants enrolled in the CHCs provided blood samples, which were immediately refrigerated and transferred under cold conditions on the same day to the Vanderbilt Epidemiology Center’s Biorepository for processing and storage at −80 °C. The SCCS study protocol was approved by the institutional review boards at Vanderbilt University and Meharry Medical College. The current study utilized completely de-identified data only.

The SCCS participants have been followed through data linkages to state cancer registries and/or the National Death Index (RRID:SCR_016369). To select incident prostate cancer cases and their matched controls, we first screened Black American males without any prior cancer history who provided blood samples at baseline. Among them, we identified a total of 195 incident prostate cancer cases (defined by the International Classification of Diseases, 10^th^ Revision: C61) with stored blood specimens. Controls were randomly selected among blood donors without any cancers, as ascertained through self-reports and data linkages, and were individually matched to cases at a 2:1 ratio based on enrollment age ( ± 2 years), enrollment sites (CHC of study enrollment), and blood collection date ( ± 6 months). In cases where few controls were available under the matching criteria, we allowed a 1:1 case-control ratio. The final analytic sample comprised 195 incident prostate cancer cases and 379 matched controls among Black American males.

### Metabolic profiling

Using pre-diagnostic plasma samples collected at baseline, a global, semi-quantitative metabolomics assay was performed by Metabolon (Morrisville, NC, USA; the Global Discovery Panel), which is capable of detecting 1503 circulating compounds, including more than 1220 named metabolites. The platform and comprehensive assay protocol have been documented elsewhere [26, 27]. In brief, ultra-high-performance liquid chromatography and gas chromatography coupled with tandem mass spectrometry were employed for measurement. The mass spectral peaks were searched in the latest chemical reference library to identify the corresponding metabolite and its relative quantity. Metabolites with known identities were grouped into eight super pathways: amino acids, carbohydrates, cofactors and vitamins, energy, lipids, nucleotides, peptides, and xenobiotics. Plasma samples of the selected case-control pairs were randomly positioned adjacent to each other inside the same assay batch. Laboratory personnel were blinded to the details of the samples.

### Statistical analysis

All metabolites were log-transformed and scaled by the mean and standard deviation (SD). Metabolites missing in ≥80% of participants were excluded, while those with values below the detection threshold were assigned half of the minimum detectable value in the dataset. Baseline characteristics between cases and controls were compared using a *t*-test for continuous variables and a Chi-square test for categorical data. Multivariable-adjusted conditional logistic regression was used to estimate odds ratios (ORs) and 95% confidence intervals (95% CIs) for the risk of developing prostate cancer associated with a 1-SD increase in log-metabolites. Covariates were selected based on literature review of potential risk factors for prostate cancer, including enrollment age (continuous, years), smoking status (current, former, never), educational attainment ( < 12 years, ≥ 12 years), household income ( < $25,000, ≥ $25,000), alcohol consumption (none, moderate, heavy; defined by 0, >0–28 gram/day of ethanol intake, and >28 gram/day of ethanol intake, respectively), marital status (living alone, living with a partner), body mass index (continuous, kg/m^2^), history of diabetes (no, yes), intake of aspirin (no, yes), and intake of vitamin supplement (no, yes). In addition, fasting status (no, yes) and blood processing time (continuous, hours from blood collection to processing) were further included in the model. Metabolites significantly associated with the development of prostate cancer were initially identified (i.e., nominal *p* < 0.01) and subsequently evaluated using stepwise regression to determine those showing independent effects on prostate cancer risk. False discovery rates (FDRs) were estimated using the Benjamini–Hochberg procedure to account for multiple comparisons [28]. To explore distinct metabolic phenotypes potentially linked primarily to aggressive prostate cancer, stratified analyses were conducted based on disease aggressiveness (aggressive vs. non-aggressive). Aggressive prostate cancer was defined as cancer stage T3/T4 or a Gleason score of ≥7 [29], indicating intermediate-to-high-grade and locally advanced forms. Moreover, we further evaluated potential effect modification by smoking status (current vs. former/never) and the time interval between blood collection and prostate cancer diagnosis ( < 5 vs. ≥5 years). Interactions were tested by likelihood ratio tests. All statistical analyses were performed using SAS 9.4 (SAS Institute, Cary, NC, USA, RRID:SCR_008567).

## Results

The mean age at enrollment for the 195 incident prostate cancer cases and 379 controls was 55 years for both groups. The mean age at prostate cancer diagnosis was 59.2 years, with cancer developing approximately 49 months after enrollment. The median duration from blood draw to prostate cancer diagnosis was 4.0 years, with an interquartile range of 2.0 to 6.0 years. Notably, about 83% of the study participants reported an annual household income of less than $25,000. Compared to controls, cases were more likely to be former smokers and married (*p* < 0.05); however, no statistically significant differences were observed in socioeconomic status, lifestyle characteristics, or preclinical conditions between cases and controls (Table 1).

**Table 1:** Baseline characteristics of study population.

	Case^a^ (*n* = 195)	Control (*n* = 379)	*p*-value
Age, mean (SD)	55.3 (5.8)	55.0 (5.8)	0.54
Education			
< 12 years	73 (37.4)	151 (39.8)	0.58
≥ 12 years	122 (62.6)	228 (60.2)	
Household income			
< $25,000	160 (82.1)	320 (84.4)	0.47
≥ $25,000	35 (17.9)	59 (15.6)	
Smoking status			
Never	37 (19.0)	83 (21.9)	0.022
Former	61 (31.3)	79 (20.8)	
Current	97 (49.7)	217 (57.3)	
Alcohol consumption^b^			
None	70 (35.9)	124 (32.7)	0.73
Moderate	68 (34.9)	142 (37.5)	
Heavy	57 (29.2)	113 (29.8)	
Marital status^c^			
Living alone	118 (60.5)	267 (70.5)	0.016
Living with a partner	77 (39.5)	112 (29.5)	
History of diabetes			
No	155 (79.5)	292 (77.0)	0.50
Yes	40 (20.5)	87 (23.0)	
Intake of aspirin^d^			
No	164 (84.1)	315 (83.1)	0.76
Yes	31 (15.9)	64 (16.9)	
Intake of supplement^e^			
No	128 (65.6)	261 (68.9)	0.43
Yes	67 (34.4)	118 (31.1)	
Body mass index (kg/m^2^), mean (SD)	28.3 (6.1)	28.0 (6.3)	0.62

Fasting status and blood processing time were included as covariates in multivariable models; neither exhibited a statistically significant difference between cases and controls (fasting status, *p* = 0.33; processing time, *p* = 0.41).

^a^The mean age at cancer diagnosis was 59.2 years, with a median time interval of 4.0 years from enrollment to cancer diagnosis (interquartile range of 2 to 6 years).

^b^Defined based on the US Dietary Guidelines for Americans 2015-2020: >0–28 g/d for moderate and >28 g/d for heavy consumption.

^c^Defined as living alone, encompassing participants who were separated, divorced, widowed, single, or never married, and living with a partner, including those who were married or cohabiting with a spouse.

^d^Regular consumption of low-dose or standard aspirin.

^e^Intake of multivitamins, vitamin E, mineral or herbal supplements, and other nutritional supplements regularly.

After adjustment for potential covariates, 26 metabolites showed a significant association with incident prostate cancer, showing a nominal *p* < 0.01 (Table 2). These metabolites were from super pathways of amino acids (S-adenosylhomocysteine (SAH) and 5-methylthioadenosine (MTA)), cofactors and vitamins (Pantoate), lipids (N-palmitoyl-heptadecasphingosine (d17:1/16:0)*, Linoleoyl-linoleoyl-glycerol (18:2/18:2) [1]*, 14-HDoHE/17-HDoHE, 12-HETE, Palmitoleoylcholine, Glycerol 3-phosphate, 1-linoleoyl-GPI (18:2)*, 1-stearoyl-2-linoleoyl-GPI (18:0/18:2), Chenodeoxycholic acid sulfate (1), Isoursodeoxych olate, Sphingomyelin (d18:1/20:0, d16:1/22:0)*, Sphingomyelin (d17:1/16:0, d18:1/15:0, d16:1/17:0)*, Sphingomyelin (d18:1/17:0, d17:1/18:0, d19:1/16:0), and Sphingomyelin (d18:1/19:0, d19:1/18:0)*), nucleotides (Inosine 5’-monophosphate (IMP), Dihydroorotate, and 2’-deoxyuridine), peptides (Cyclo(leu-pro), Isoleucylglycine, and Gamma-glutamyl-epsilon-lysine), xenobiotics (Trizma acetate, 2,2’-Methylenebis(6-tert-butyl-p-cresol), and Ethyl alpha-glucopyranoside), and two unnamed metabolites (X_24762 and X_12753). Their corresponding ORs per 1-SD increase ranged from 0.61 to 0.77 for inverse associations and from 1.29 to 1.47 for positive associations with prostate cancer. Given that over 1500 metabolites were examined, about 15 would be expected by chance alone when considering 0.01 individual *p*-values. After correction for multiple comparisons, all FDR-adjusted *p*-values exceeded 0.20; therefore, none of the associations were considered statistically significant in the FDR-corrected analyses.

**Table 2:** Associations of metabolites with prostate cancer risk (with nominal *p* < 0.01).

Super Pathway	Name^a^	OR (95% CI)^b^	*P*^c^	Sub Pathway
Amino Acid	S-adenosylhomocysteine (SAH)	0.67 (0.54–0.83)	0.0003	Methionine, Cysteine, SAM, Taurine Metabolism
	5-methylthioadenosine (MTA)	0.72 (0.60–0.88)	0.0011	Polyamine Metabolism
Cofactors & Vitamins	Pantoate	0.76 (0.63–0.92)	0.0059	Pantothenate and CoA Metabolism
Lipid	N-palmitoyl-heptadecasphingosine (d17:1/16:0)*	1.44 (1.12–1.86)	0.0050	Ceramides
	Linoleoyl-linoleoyl-glycerol (18:2/18:2) [1]*	0.76 (0.63–0.93)	0.0063	Diacylglycerol
	14-HDoHE/17-HDoHE	1.33 (1.09–1.63)	0.0060	Docosanoid
	12-HETE	1.39 (1.14–1.69)	0.0013	Eicosanoid
	Palmitoleoylcholine	0.76 (0.62–0.93)	0.0090	Fatty Acid Metabolism (Acyl Choline)
	Glycerol 3-phosphate	0.70 (0.57–0.86)	0.0008	Glycerolipid Metabolism
	1-linoleoyl-GPI (18:2)*	0.69 (0.56–0.86)	0.0007	Lysophospholipid
	1-stearoyl-2-linoleoyl-GPI (18:0/18:2)	0.71 (0.58–0.87)	0.0009	Phosphatidylinositol (PI)
	Chenodeoxycholic acid sulfate (1)	0.72 (0.58–0.89)	0.0025	Primary Bile Acid Metabolism
	Isoursodeoxycholate	0.77 (0.64–0.93)	0.0071	Secondary Bile Acid Metabolism
	Sphingomyelin (d18:1/20:0, d16:1/22:0)*	1.32 (1.07–1.63)	0.0094	Sphingomyelins
	Sphingomyelin (d17:1/16:0, d18:1/15:0, d16:1/17:0)*	1.37 (1.12–1.66)	0.0018	Sphingomyelins
	Sphingomyelin (d18:1/17:0, d17:1/18:0, d19:1/16:0)	1.35 (1.10–1.66)	0.0047	Sphingomyelins
	Sphingomyelin (d18:1/19:0, d19:1/18:0)*	1.36 (1.09–1.70)	0.0071	Sphingomyelins
Nucleotide	Inosine 5’-monophosphate (IMP)	1.35 (1.09–1.66)	0.0058	Purine Metabolism, Xanthine/Inosine containing
	Dihydroorotate	0.72 (0.58–0.88)	0.0018	Pyrimidine Metabolism, Orotate containing
	2’-deoxyuridine	0.70 (0.57–0.87)	0.0014	Pyrimidine Metabolism, Uracil containing
Peptide	Cyclo(leu-pro)	0.75 (0.61–0.93)	0.0090	Dipeptide
	Isoleucylglycine	1.29 (1.06–1.56)	0.0096	Dipeptide
	Gamma-glutamyl-epsilon-lysine	1.47 (1.19–1.82)	0.0004	Gamma-glutamyl Amino Acid
Xenobiotics	Trizma acetate	1.34 (1.07–1.67)	0.0099	Chemical
	2,2’-Methylenebis(6-tert-butyl-p-cresol)	0.61 (0.42–0.88)	0.0077	Chemical
	Ethyl alpha-glucopyranoside	0.71 (0.57–0.89)	0.0024	Food Component/Plant
Unknown	X_24762	0.73 (0.59–0.91)	0.0041	-
	X_12753	0.74 (0.61–0.90)	0.0021	-

^a^1 standard deviation (SD) increase in natural logarithm-transformed standardized metabolite levels.

^b^Estimated using multivariable conditional logistic regression adjusted for enrollment age (years), smoking status (current, former, never), education ( < 12 years, ≥ 12 years), household income ( < $25,000, ≥ $25,000), alcohol consumption (none, moderate, heavy), marital status (living alone, living with a partner), body mass index (continuous, kg/m^2^), history of diabetes (yes, no), intake of aspirin (no, yes), intake of supplement (no, yes), blood fasting status (no, yes), blood processing time (continuous, hours).

^c^Raw *p*-values in multivariable conditional logistic regression models. All false discovery rate (FDR)-adjusted *p*-values were above 0.20.

Among the 26 metabolites linked to incident prostate cancer, seven were determined as potential independent biomarkers for prostate cancer in Black American males using stepwise selection (Table 3). Three metabolites were associated with increased risk of prostate cancer: 14-HDoHE/17-HDoHE (OR [95% CI] per 1-SD increment = 1.46 [1.17–1.83]), sphingomyelin (d17:1/16:0, d18:1/15:0, d16:1/17:0)* (1.28 [1.03–1.59]), and gamma-glutamyl-epsilon-lysine (1.45 [1.14–1.85]). Meanwhile, the other four metabolites showed associations with decreased risk of developing prostate cancer among Black Americans: SAH (0.69 [0.55–0.88]), 1-linoleoyl-GPI (18:2)* (0.73 [0.57–0.92]), 2,2’-Methylenebis(6-tert-butyl-p-cresol) (0.62 [0.40-0.95]), and X_24762 (0.79 [0.62–1.00]).

**Table 3:** Metabolites independently associated with prostate cancer risk: stepwise selection.

Super Pathway	Name^a^	OR (95% CI)^b^	*P*^c^	Sub Pathway
Amino Acid	S-adenosylhomocysteine (SAH)	0.69 (0.55–0.88)	0.0024	Methionine, Cysteine, SAM, Taurine Metabolism
Lipid	14-HDoHE/17-HDoHE	1.46 (1.17–1.83)	0.0009	Docosanoid
	1-linoleoyl-GPI (18:2)*	0.73 (0.57–0.92)	0.0086	Lysophospholipid
	Sphingomyelin (d17:1/16:0, d18:1/15:0, d16:1/17:0)*	1.28 (1.03–1.59)	0.0235	Sphingomyelins
Peptide	Gamma-glutamyl-epsilon-lysine	1.45 (1.14–1.85)	0.0026	Gamma-glutamyl Amino Acid
Xenobiotics	2,2’-Methylenebis(6-tert-butyl-p-cresol)	0.62 (0.40–-0.95)	0.0265	Chemical
Unknown	X_24762	0.79 (0.62–1.00)	0.0489	-

^a^1 standard deviation (SD) increase in natural logarithm-transformed standardized metabolite levels.

^b^Estimated using multivariable conditional logistic regression, including all listed metabolites in a single model, with adjustment for enrollment age (years), smoking status (current, former, never), education ( < 12 years, ≥ 12 years), household income ( < $25,000, ≥ $25,000), alcohol consumption (none, moderate, heavy), marital status (living alone, living with a partner), body mass index (continuous, kg/m^2^), history of diabetes (yes, no), intake of aspirin (no, yes), intake of supplement (no, yes), blood fasting status (no, yes), blood processing time (continuous, hours).

^c^Raw *p*-values in multivariable conditional logistic regression models.

Upon stratification by the aggressiveness of prostate cancer (Table 4), SAH and 14-HDoHE/17-HDoHE revealed a nominally significant association exclusively with aggressive prostate cancer, with respective ORs (95% CIs) per 1-SD increment of 0.52 (0.36–0.76) and 1.60 (1.14–2.25). On the contrary, five other metabolites, including 1-linoleoyl-GPI (18:2)* (0.59 [0.43–0.81]), sphingomyelin (d17:1/16:0, d18:1/15:0, d16:1/17:0)* (1.65 [1.24–2.21]), gamma-glutamyl-epsilon-lysine (1.66 [1.21–2.29]), 2,2’-Methylenebis(6-tert-butyl-p-cresol) (0.51 [0.30–0.87]), and X_24762 (0.65 [0.48–0.88]), were only linked to non-aggressive prostate cancer. The associations of SAH, sphingomyelin (d17:1/16:0, d18:1/15:0, d16:1/17:0)*, gamma-glutamyl-epsilon-lysine, and X_24762 with prostate cancer risk remained nominally significant regardless of smoking status. The ORs (95% CIs) per 1-SD increment for current smokers compared to former/never smokers were 0.60 (0.45–0.82) vs. 0.77 (0.59–0.99) for SAH, 1.30 (1.00–1.68) vs. 1.43 (1.07–1.91) for sphingomyelin (d17:1/16:0, d18:1/15:0, d16:1/17:0)*, 1.37 (1.05–1.80) vs. 1.39 (1.05–1.85) for gamma-glutamyl-epsilon-lysine, and 0.77 (0.59–0.99) vs. 0.69 (0.50–0.95) for X_24762. The overall patterns of initial associations did not differ by the time interval between blood collection and the diagnosis of prostate cancer; these findings were consistent even after excluding cases who developed prostate cancer within the first year of enrollment and their matched controls (data not shown). The power to evaluate any interactions was limited—no apparent interaction was observed between the seven selected metabolites and stratification characteristics (all *p*-interaction >0.05).

**Table 4:** Association of selected metabolites with prostate cancer risk: stratified analyses by aggressiveness, smoking status, and time interval from enrollment to cancer diagnosis.

Super Pathway	Name^a^	OR (95% CI) ^b^	*P*^c^	OR (95% CI)^b^	*P*^c^	*P*_interaction_
		**Aggressive,** ***n*** = **252**		**Non-Aggressive,** ***n*** = **322**		
		Case *n* = 85 / Controls *n* = 167		Case *n* = 110 / Controls *n* = 212		
Amino Acid	S-adenosylhomocysteine (SAH)	0.52 (0.36–0.76)	0.0006	0.82 (0.61–1.11)	0.1905	0.057
Lipid	14-HDoHE/17-HDoHE	1.60 (1.14–2.25)	0.0067	1.17 (0.88–1.56)	0.2783	0.119
	1-linoleoyl-GPI (18:2)*	0.92 (0.67–1.26)	0.5996	0.59 (0.43–0.81)	0.0010	0.088
	Sphingomyelin (d17:1/16:0, d18:1/15:0, d16:1/17:0)*	1.24 (0.91–1.68)	0.1775	1.65 (1.24–2.21)	<0.001	0.096
Peptide	Gamma-glutamyl-epsilon-lysine	1.36 (0.99–1.86)	0.0557	1.66 (1.21–2.29)	0.0017	0.310
Xenobiotics	2,2’-Methylenebis(6-tert-butyl-p-cresol)	0.75 (0.42–1.32)	0.3176	0.51 (0.30–0.87)	0.0131	0.603
Unknown	X_24762	0.80 (0.57–1.13)	0.1965	0.65 (0.48–0.88)	0.0054	0.327
		**Current Smokers,** ***n*** = **314**		**Former/Never Smokers,** ***n*** = **260**		
		Case *n* = 97 / Controls *n* = 217		Case *n* = 98 / Controls *n* = 162		
Amino Acid	S-adenosylhomocysteine (SAH)	0.60 (0.45–0.82)	0.0010	0.77 (0.59–0.99)	0.0443	0.533
Lipid	14-HDoHE/17-HDoHE	1.20 (0.91–1.58)	0.1904	1.38 (1.06–1.79)	0.0172	0.371
	1-linoleoyl-GPI (18:2)*	0.82 (0.62–1.09)	0.1760	0.66 (0.50–0.86)	0.0025	0.412
	Sphingomyelin (d17:1/16:0, d18:1/15:0, d16:1/17:0)*	1.30 (1.00–1.68)	0.0517	1.43 (1.07–1.91)	0.0170	0.490
Peptide	Gamma-glutamyl-epsilon-lysine	1.37 (1.05–1.80)	0.0210	1.39 (1.05–1.85)	0.0215	0.918
Xenobiotics	2,2’-Methylenebis(6-tert-butyl-p-cresol)	0.83 (0.65–1.07)	0.1426	0.89 (0.69–1.15)	0.3889	0.689
Unknown	X_24762	0.77 (0.59–0.99)	0.0436	0.69 (0.50–0.95)	0.0235	0.500
		**< 5 Years,** ***n*** = **315**		**≥ 5 Years,** ***n*** = **259**		
		Case *n* = 108 / Controls *n* = 207		Case *n* = 87 / Controls *n* = 172		
Amino Acid	S-adenosylhomocysteine (SAH)	0.76 (0.59–0.99)	0.0438	0.50 (0.33–0.74)	<0.001	0.076
Lipid	14-HDoHE/17-HDoHE	1.28 (0.98–1.68)	0.0689	1.49 (1.05–2.12)	0.0264	0.634
	1-linoleoyl-GPI (18:2)*	0.77 (0.58–1.02)	0.0718	0.55 (0.39–0.80)	0.0014	0.188
	Sphingomyelin (d17:1/16:0, d18:1/15:0, d16:1/17:0)*	1.30 (0.99–1.71)	0.0627	1.49 (1.10–2.01)	0.0103	0.381
Peptide	Gamma-glutamyl-epsilon-lysine	1.49 (1.11–2.00)	0.0075	1.46 (1.03–2.05)	0.0325	0.797
Xenobiotics	2,2’-Methylenebis(6-tert-butyl-p-cresol)	0.67 (0.41–1.08)	0.0970	0.56 (0.30–1.02)	0.0593	0.659
Unknown	X_24762	0.67 (0.49–0.91)	0.0097	0.75 (0.53–1.05)	0.0944	0.658

^a^1 standard deviation (SD) increase in natural logarithm-transformed standardized metabolite levels.

^b^Adjusted for enrollment age (years), smoking status (current, former, never), education ( < 12 years, ≥ 12 years), household income ( < $25,000, ≥ $25,000), alcohol consumption (none, moderate, heavy), marital status (living alone, living with a partner), body mass index (continuous, kg/m^2^), history of diabetes (yes, no), intake of aspirin (no, yes), intake of supplement (no, yes), blood fasting status (no, yes), blood processing time (continuous, hours).

^c^Raw *p*-values in multivariable conditional logistic regression models.

## Discussion

In this prospective metabolomics study of low-income Black American males, we identified seven metabolites independently associated with prostate cancer risk, including two linked to its aggressive forms, after accounting for the potential risk factors for prostate cancer. The overall association did not differ by smoking status and the time interval between blood draw and cancer diagnosis. Although none of the associations remained significant after correcting for the multiple comparisons, the findings raise hypotheses toward potential metabolic biomarkers that may contribute to the burden of prostate cancer among Black Americans.

A recent systematic review of 29 prospective metabolomics studies identified 42 metabolites, including citrate, glycine, glutamate, gamma-glutamylphenyalanine, palmitic acid, and linolenic acid, as repeatedly associated with prostate cancer across various cohort studies, suggesting their potential as risk assessment or predictive biomarkers for the disease [30]. Of these, only three metabolites, including 5-methylthioadenosine (MTA), 1-stearoyl-2-linoleoyl-GPI (18:0/18:2)—also referred to as oleoyl-linoleoyl-glycerophosphoinositol (GPI)—and 2’-deoxyuridine, each exhibited a nominally significant association with prostate cancer risk (nominal *p* < 0.01) in the current study. Yet, upon the application of stepwise selection, none emerged as potential independent biomarkers for prostate cancer in Black American males. The discrepancy may be partly attributed to racial differences, given that the majority of previous metabolomics research has predominantly involved males of European ancestry. Although several previous studies have investigated specific metabolites linked to prostate cancer risk, the overall epidemiological evidence remains inconclusive, particularly for Black Americans. Our understanding is still limited concerning the metabolic characteristics associated with the onset and/or progression of prostate cancer in Black American males, who are at heightened risk of developing and dying from the disease. This prospective metabolomics study of Black prostate cancer cases and matched controls suggested metabolites of amino acids (SAH), lipids (14-HDoHE/17-HDoHE, 1-linoleoyl-GPI (18:2)*, and sphingomyelin (d17:1/16:0, d18:1/15:0, d16:1/17:0)*), peptides (gamma-glutamyl-epsilon-lysine), and xenobiotics (2,2’-Methylenebis(6-tert-butyl-p-cresol)) as potential biomarkers for prostate cancer risk among Black Americans.

Our findings indicated a possible inverse association between SAH and prostate cancer risk, particularly pronounced in aggressive forms, among Black Americans, with similar associations observed across categories of smoking status and the time span between blood collection and diagnosis. SAH, a metabolic precursor of homocysteine and a strong inhibitor of DNA methyltransferases, has been implicated in the pathogenesis of various chronic diseases, including cardiovascular events and kidney disease [31]. However, its role in carcinogenesis remains poorly understood and even contradictory: plasma SAH levels showed an inverse association with colorectal adenoma among men, but a strong positive association among women [31], whereas elevated circulating SAH levels were significantly linked to poorer prognosis in hepatocellular carcinoma [32]. Currently, limited evidence exists regarding the molecular mechanisms linking SAH to cancer risk, as well as epidemiological studies on the SAH-prostate cancer association. Given the link between SAH, folate metabolism, and prostate cancer [33], the observed inverse association between SAH and prostate cancer risk may be explained by SAH’s potential role in regulating the methylation of tumor suppressor genes or oncogenes governed by one-carbon metabolism [34]. Further large-scale, prospective studies, integrated with mechanistic investigations, are required to validate our findings and elucidate the biological mechanisms underlying SAH’s effects on prostate carcinogenesis.

This study identified three metabolites of amino acids linked to prostate cancer risk among Black American males at a nominal significance level, showing a positive association with 14-HDoHE/17-HDoHE and sphingomyelin (d17:1/16:0, d18:1/15:0, d16:1/17:0)* while revealing an inverse association with 1-linoleoyl-GPI (18:2)*. 14-HDoHE/17-HDoHE are metabolic derivatives of docosahexaenoic acid (DHA), an omega-3 fatty acid implicated in cardiovascular disease and cardiometabolic outcomes [35, 36]. While several in vitro and animal studies suggest potential anti-cancer properties of these metabolites [37, 38], the molecular processes underlying DHA or omega-3 metabolism remain poorly understood in prostate carcinogenesis. Furthermore, epidemiological evidence, although limited, indicates a strong positive linear association between blood concentrations of omega-3 fatty acids—specifically DHA and docosapentaenoic acid—and prostate cancer risk, even high-grade prostate cancer, implying their involvement in promoting prostate carcinogenesis [39, 40]. In line with these findings, we observed a positive association of DHA metabolites, 14-HDoHE/17-HDoHE, with prostate cancer risk in Black Americans, particularly for the aggressive forms of the disease. Given the possible multifaceted role of DHA and omega-3 fatty acids suggested thus far, future metabolomics research, accompanied by in-depth mechanistic investigations, will enhance the understanding of the complex and potentially dual nature of omega-3 fatty acids in prostate carcinogenesis. Sphingolipids play a crucial role in various biological mechanisms—e.g., cell death and proliferation—which have been widely implicated in various types of cancer, including prostate cancer [41]. We found a nominally significant association between several metabolites of sphingomyelins and prostate cancer, with sphingomyelin (d17:1/16:0, d18:1/15:0, d16:1/17:0)* being possible as an independent biomarker linked to prostate carcinogenesis in Black Americans. In light of the potential value of sphingolipid-based chemotherapeutics [42, 43], our findings could provide novel insight into the sphingolipid metabolism of Black Americans in the context of personalized prostate cancer treatment. Compared to other lipid metabolites, 1-linoleoyl-GPI (18:2)* was underrepresented in previous studies; however, a recent two-sample Mendelian randomization study among European descendants indicated a significant protective effect of 1-linoleoyl-GPI (18:2)* against breast cancer risk [44], which parallels our finding of inverse association with prostate cancer.

Gamma-glutamyl-epsilon-lysine, one of the gamma-glutamyl-peptides, exhibited a nominally significant positive association with the risk of prostate cancer in Black Americans—this association remained consistent irrespective of smoking status and the time interval from blood draw and cancer diagnosis. Similarly, previous metabolomics investigations based on the Alpha-Tocopherol, Beta-Carotene Cancer Prevention (ATBC) Study reported a significantly increased risk of lethal prostate cancer and prostate cancer-specific mortality linked to various gamma-glutamyl-peptides among Caucasian male smokers [45, 46], while gamma-glutamyl-epsilon-lysine showed a non-significant positive association. Gamma-glutamyl-peptides are byproducts of glutathione metabolism, regulated by gamma-glutamyltransferase (GGT), a key factor in drug resistance, invasion, and tumor progression [47]. Higher concentrations of circulating GGT have been associated with a higher risk of prostate cancer [48, 49]. Black American males have been reported to have particularly high GGT levels [50], which may partially contribute to their disproportionate burden of prostate cancer.

Our findings suggest that SAH and 14-HDoHE/17-HDoHE could serve as potential biomarkers for aggressive prostate cancer among Black Americans. Some studies of European Americans reported metabolic profiles related to aggressive forms of prostate cancer. For instance, the ATBC study found inverse associations between energy and lipid metabolites (e.g., inositol-1-phosphate, glycerophospholipids, alpha-ketoglutarate, and citrate) and aggressive prostate cancer [15]. In the European Prospective Investigation into Cancer and Nutrition (EPIC) study, men with three distinct metabolite profiles—characterized by elevated levels of (1) phosphatidylcholines and hydroxysphingomyelins, (2) acylcarnitines C18:1 and C18:2, glutamate, ornithine, and taurine, or (3) lysophosphatidylcholines—demonstrated a decreased risk of advanced-stage prostate cancer at the time of diagnosis [51]. Another EPIC study reported that dimethylglycine and 3-hydroxybenzaldehyde were associated with advanced prostate cancer [19]. The differential associations across disease aggressiveness and cancer stages may suggest the potential heterogeneity in metabolic alterations related to prostate cancer progression, highlighting the complex and dynamic nature of prostate cancer metabolism. This would provide crucial insights into the pathogenesis of prostate cancer among Black American males who are more likely to be diagnosed with aggressive prostate cancer. Given that the association of SAH with prostate cancer did not vary by the time interval between blood draw and diagnosis, as well as smoking status, its prospective application as a metabolic indicator for risk stratification and prostate cancer prediction may be implicated.

The major strengths of our study include a specifically targeted cohort of Black American males, who are disproportionately affected by prostate cancer yet are underrepresented in prior research. Our prospective design, incorporating pre-diagnostic blood samples and detailed baseline information, minimized the possibility of reverse causation and confounding bias. The application of comprehensive untargeted metabolomic profiling, covering over 1500 metabolites, enabled an extensive assessment of potential metabolic indicators for Black Americans and provided insights for future investigations aimed at elucidating molecular pathways linked to prostate carcinogenesis. Meanwhile, several limitations should be acknowledged. First, our sample size was somewhat limited—none of the findings achieved statistical significance upon accounting for multiple comparisons (FDR < 0.05). Insufficient power could also lead to the oversight of putative moderate-effect metabolites and limit our ability to implement more rigorous clinical definitions of disease aggressiveness, such as a Gleason score ≥8. Further studies with expanded sample sizes are necessary to confirm the findings of this study and enable more comprehensive exploration. Second, the unavailability of serial blood samples (i.e., single-point estimation) meant that we could not measure metabolite levels in the same individuals over time and ascertain within-individual repeatability of the markers. Third, we could not rule out the potential for residual confounding due to unmeasured or inadequately measured risk factors. Larger prospective investigations coupled with mechanistic analysis will address the limitations and may enhance our understanding of the pathophysiological factors contributing to prostate cancer risk and disparities.

## Conclusion

This comprehensive metabolomic study of Black American males identified several metabolites nominally significantly and independently associated with prostate cancer risk, including 14-HDoHE/17-HDoHE, sphingomyelin (d17:1/16:0, d18:1/15:0, d16:1/17:0), and gamma-glutamyl-epsilon-lysine, which showed positive associations, whereas SAH, 1-linoleoyl-GPI (18:2), and 2,2’-Methylenebis(6-tert-butyl-p-cresol) exhibited inverse associations. These findings suggest the potential metabolic involvement in one-carbon, DHA/omega-3, sphingolipid, and glutathione pathways toward prostate carcinogenesis among Black Americans. Further research warrants validating our findings and investigating whether the varying levels of these metabolites may translate to the disproportionate burden of prostate cancer among Black Americans.

## Data Availability

Data used in the present study can be requested through the Southern Community Cohort Study online request system (https://ors.southerncommunitystudy.org/).

## References

[1] Kratzer TB, Mazzitelli N, Star J, Dahut WL, Jemal A, Siegel RL. Prostate cancer statistics. 2025;75:485–97. 10.3322/caac.70028 PMC12593258 40892160

[2] American Cancer Society. Cancer Facts & Figures 2025. Atlanta: American Cancer Society; 2025.

[3] American Cancer Society. Cancer Facts & Figures for African Americans 2019–2021. Atlanta: American Cancer Society; 2019.

[4] Lillard JW, Moses KA, Mahal BA, George DJ. Racial disparities in Black men with prostate cancer: a literature review. Cancer. 2022;128:3787–95. 36066378 10.1002/cncr.34433 PMC9826514

[5] Rebbeck TR. Prostate cancer disparities by race and ethnicity: from nucleotide to neighborhood. Cold Spring Harb Perspect Med. 2018;8:a030387. 29229666 10.1101/cshperspect.a030387 PMC6120694

[6] Moses KA, Sprenkle PC, Bahler C, Box G, Carlsson SV, Catalona WJ, et al. NCCN guidelines® insights: prostate cancer early detection, version 1.2023. J Natl Compr Canc Netw. 2023;21:236–46. 36898362 10.6004/jnccn.2023.0014

[7] Giaquinto AN, Miller KD, Tossas KY, Winn RA, Jemal A, Siegel RL. Cancer statistics for African American/Black People 2022. CA Cancer J Clin. 2022;72:202–29. 35143040 10.3322/caac.21718

[8] Dovey ZS, Nair SS, Chakravarty D, Tewari AK. Racial disparity in prostate cancer in the African American population with actionable ideas and novel immunotherapies. Cancer Rep (Hoboken). 2021;4:e1340. 33599076 10.1002/cnr2.1340 PMC8551995

[9] Gómez-Cebrián N, Poveda JL, Pineda-Lucena A, Puchades-Carrasco L. Metabolic phenotyping in prostate cancer using multi-omics approaches. Cancers (Basel). 2022;14:596. 35158864 10.3390/cancers14030596 PMC8833769

[10] Kelly RS, Vander Heiden MG, Giovannucci E, Mucci LA. Metabolomic biomarkers of prostate cancer: prediction, diagnosis, progression, prognosis, and recurrence. Cancer Epidemiol Biomark Prev. 2016;25:887–906. 10.1158/1055-9965.EPI-15-1223 PMC4891222 27197278

[11] Larkin JR, Anthony S, Johanssen VA, Yeo T, Sealey M, Yates AG, et al. Metabolomic biomarkers in blood samples identify cancers in a mixed population of patients with nonspecific symptoms. Clin Cancer Res. 2022;28:1651–61. 34983789 10.1158/1078-0432.CCR-21-2855 PMC7613224

[12] Gómez-Cebrián N, Rojas-Benedicto A, Albors-Vaquer A, López-Guerrero JA, Pineda-Lucena A, Puchades-Carrasco L. Metabolomics contributions to the discovery of prostate cancer biomarkers. Metabolites. 2019;9:48. 30857149 10.3390/metabo9030048 PMC6468766

[13] Penney KL, Tyekucheva S, Rosenthal J, El Fandy H, Carelli R, Borgstein S, et al. Metabolomics of prostate cancer Gleason score in tumor tissue and serum. Mol Cancer Res. 2021;19:475–84. 33168599 10.1158/1541-7786.MCR-20-0548 PMC8369519

[14] De Vogel S, Ulvik A, Meyer K, Ueland PM, Nygård O, Vollset SE, et al. Sarcosine and other metabolites along the choline oxidation pathway in relation to prostate cancer: a large nested case-control study within the JANUS cohort in Norway. Int J Cancer. 2014;134:197–206. 23797698 10.1002/ijc.28347

[15] Mondul AM, Moore SC, Weinstein SJ, Karoly ED, Sampson JN, Albanes D. Metabolomic analysis of prostate cancer risk in a prospective cohort: the alpha-tocopherol, beta-carotene cancer prevention (ATBC) study. Int J Cancer. 2015;137:2124–32. 25904191 10.1002/ijc.29576 PMC4537663

[16] Huang J, Mondul AM, Weinstein SJ, Koutros S, Derkach A, Karoly E, et al. Serum metabolomic profiling of prostate cancer risk in the prostate, lung, colorectal, and ovarian cancer screening trial. Br J Cancer. 2016;115:1087–95. 27673363 10.1038/bjc.2016.305 PMC5117796

[17] Lin X, Lécuyer L, Liu X, Triba MN, Deschasaux-Tanguy M, Demidem A, et al. Plasma metabolomics for discovery of early metabolic markers of prostate cancer based on ultra-high-performance liquid chromatography-high resolution mass spectrometry. Cancers (Basel). 2021;13:3140. 34201735 10.3390/cancers13133140 PMC8268247

[18] Schmidt JA, Fensom GK, Rinaldi S, Scalbert A, Appleby PN, Achaintre D, et al. Patterns in metabolite profile are associated with risk of more aggressive prostate cancer: a prospective study of 3,057 matched case–control sets from EPIC. Int J Cancer. 2020;146:720–30. 30951192 10.1002/ijc.32314 PMC6916595

[19] Schmidt JA, Fensom GK, Rinaldi S, Scalbert A, Appleby PN, Achaintre D, et al. Pre-diagnostic metabolite concentrations and prostate cancer risk in 1077 cases and 1077 matched controls in the European Prospective Investigation into Cancer and Nutrition. BMC Med. 2017;15:122. 28676103 10.1186/s12916-017-0885-6 PMC5497352

[20] Röhnisch HE, Kyrø C, Olsen A, Thysell E, Hallmans G, Moazzami AA. Identification of metabolites associated with prostate cancer risk: a nested case-control study with long follow-up in the Northern Sweden Health and Disease Study. BMC Med. 2020;18:187. 32698845 10.1186/s12916-020-01655-1 PMC7376662

[21] Ravindran A, Piyarathna DWB, Gohlke J, Putluri V, Soni T, Lloyd S, et al. Lipid alterations in African American men with prostate cancer. Metabolites. 2021;12:8. 35050130 10.3390/metabo12010008 PMC8779756

[22] Teas J, Cunningham JE, Fowke JH, Nitcheva D, Kanwat CP, Boulware RJ, et al. Urinary estrogen metabolites, prostate specific antigen, and body mass index among African–American men in South Carolina. Cancer Detect Prev. 2005;29:494–500. 16289388 10.1016/j.cdp.2005.08.004

[23] Richards Z, Batai K, Farhat R, Shah E, Makowski A, Gann PH, et al. Prostatic compensation of the vitamin D axis in African American men. JCI Insight. 2017;2:e91054. 28138564 10.1172/jci.insight.91054 PMC5256134

[24] Signorello LB, Hargreaves MK, Blot WJ. The Southern Community Cohort Study: investigating health disparities. J Health Care Poor Underserved. 2010;21:26–37. 20173283 10.1353/hpu.0.0245 PMC2940058

[25] Signorello LB, Hargreaves MK, Steinwandel MD, Zheng W, Cai Q, Schlundt DG, et al. Southern community cohort study: establishing a cohort to investigate health disparities. J Natl Med Assoc. 2005;97:972–9. 16080667 PMC2569308

[26] Evans AM, DeHaven CD, Barrett T, Mitchell M, Milgram E. Integrated, nontargeted ultrahigh performance liquid chromatography/electrospray ionization tandem mass spectrometry platform for the identification and relative quantification of the small-molecule complement of biological systems. Anal Chem. 2009;81:6656–67. 19624122 10.1021/ac901536h

[27] Evans AM, Bridgewater BR, Liu Q, Mitchell MW, Robinson RJ, Dai H, et al. High resolution mass spectrometry improves data quantity and quality as compared to unit mass resolution mass spectrometry in high-throughput profiling metabolomics. Metabolomics. 2014;4:132.

[28] Benjamini Y, Hochberg Y. Controlling the false discovery rate: a practical and powerful approach to multiple testing. J R Stat Soc Ser B Stat Methodol. 1995;57:289–300.

[29] Epstein JI, Zelefsky MJ, Sjoberg DD, Nelson JB, Egevad L, Magi-Galluzzi C, et al. A contemporary prostate cancer grading system: a validated alternative to the gleason score. Eur Urol. 2016;69:428–35. 26166626 10.1016/j.eururo.2015.06.046 PMC5002992

[30] López-Hernández Y, Andres-Lacueva C, Wishart DS, Torres-Calzada C, Martínez-Huélamo M, Almanza-Aguilera E, et al. Prostate cancer risk biomarkers from large cohort and prospective metabolomics studies: a systematic review. Transl Oncol. 2025;51:102196. 39580963 10.1016/j.tranon.2024.102196 PMC11625367

[31] Shrubsole MJ, Wagner C, Zhu X, Hou L, Loukachevitch LV, Ness RM, et al. Associations between S-adenosylmethionine, S-adenosylhomocysteine, and colorectal adenoma risk are modified by sex. Am J Cancer Res. 2014;5:458–65. 25628954 PMC4300688

[32] Wusiman M, Huang SY, Liu ZY, He TT, Fang AP, Li MC, et al. Serum S-adenosylhomocysteine, rather than homocysteine, is associated with hepatocellular carcinoma survival: a prospective cohort study. Am J Clin Nutr. 2024;120:481–90. 39025328 10.1016/j.ajcnut.2024.07.014

[33] Ducker GS, Rabinowitz JD. One-carbon metabolism in health and disease. Cell Metab. 2017;25:27–42. 27641100 10.1016/j.cmet.2016.08.009 PMC5353360

[34] Labbé DP, Zadra G, Ebot EM, Mucci LA, Kantoff PW, Loda M, et al. Role of diet in prostate cancer: the epigenetic link. Oncogene. 2015;34:4683–91. 25531313 10.1038/onc.2014.422 PMC4476943

[35] Caligiuri SPB, Parikh M, Stamenkovic A, Pierce GN, Aukema HM. Dietary modulation of oxylipins in cardiovascular disease and aging. Am J Physiol Heart Circ Physiol. 2017;313:H903–18. 28801523 10.1152/ajpheart.00201.2017

[36] Ağagündüz D, Yeşildemir Ö, Koçyiğit E, Koçak T, Özen Ünaldı BÖ, Ayakdaş G, et al. Oxylipins derived from PUFAs in cardiometabolic diseases: mechanism of actions and possible nutritional interactions. Nutrients. 2024;16:3812. 39599599 10.3390/nu16223812 PMC11597274

[37] Nakanishi M, Hanley MP, Zha R, Igarashi Y, Hull MA, Mathias G, et al. A novel bioactive derivative of eicosapentaenoic acid (EPA) suppresses intestinal tumor development in ApcΔ14/+ mice. Carcinogenesis. 2018;39:429–38. 29206907 10.1093/carcin/bgx136

[38] Bai X, Shao J, Zhou S, Zhao Z, Li F, Xiang R, et al. Inhibition of lung cancer growth and metastasis by DHA and its metabolite, RvD1, through miR-138-5p/FOXC1 pathway. J Exp Clin Cancer Res. 2019;38:479. 31783879 10.1186/s13046-019-1478-3 PMC6884860

[39] Brasky TM, Darke AK, Song X, Tangen CM, Goodman PJ, Thompson IM, et al. Plasma phospholipid fatty acids and prostate cancer risk in the SELECT trial. J Natl Cancer Inst. 2013;105:1132–41. 23843441 10.1093/jnci/djt174 PMC3735464

[40] Brasky TM, Till C, White E, Neuhouser ML, Song X, Goodman P, et al. Serum phospholipid fatty acids and prostate cancer risk: results from the prostate cancer prevention trial. Am J Epidemiol. 2011;173:1429–39. 21518693 10.1093/aje/kwr027 PMC3145396

[41] Tallima H, Azzazy HME, El Ridi R. Cell surface sphingomyelin: key role in cancer initiation, progression, and immune evasion. Lipids Health Dis. 2021;20:150. 34717628 10.1186/s12944-021-01581-y PMC8557557

[42] Voelkel-Johnson C, Norris JS, White-Gilbertson S. Interdiction of sphingolipid metabolism revisited: focus on prostate cancer. Adv Cancer Res. 2018;140:265–93. 30060812 10.1016/bs.acr.2018.04.014 PMC6460930

[43] Shaw J, Costa-Pinheiro P, Patterson L, Drews K, Spiegel S, Kester M. Novel sphingolipid-based cancer therapeutics in the personalized medicine era. Adv Cancer Res. 2018;140:327–66. 30060815 10.1016/bs.acr.2018.04.016 PMC6320700

[44] Chen Y, Xie Y, Ci H, Cheng Z, Kuang Y, Li S, et al. Plasma metabolites and risk of seven cancers: a two-sample Mendelian randomization study among European descendants. BMC Med. 2024;22:90. 38433226 10.1186/s12916-024-03272-8 PMC10910673

[45] Huang J, Mondul AM, Weinstein SJ, Derkach A, Moore SC, Sampson JN, et al. Prospective serum metabolomic profiling of lethal prostate cancer. Int J Cancer. 2019;145:3231–43. 30779128 10.1002/ijc.32218 PMC6698432

[46] Huang J, Zhao B, Weinstein SJ, Albanes D, Mondul AM. Metabolomic profile of prostate cancer-specific survival among 1812 Finnish men. BMC Med. 2022;20:362. 36280842 10.1186/s12916-022-02561-4 PMC9594924

[47] Corti A, Franzini M, Paolicchi A, Pompella A. Gamma-glutamyltransferase of cancer cells at the crossroads of tumor progression, drug resistance and drug targeting. Anticancer Res. 2010;30:1169–81. 20530424

[48] Van Hemelrijck M, Jassem W, Walldius G, Fentiman IS, Hammar N, Lambe M, et al. Gamma-glutamyltransferase and risk of cancer in a cohort of 545,460 persons - the Swedish AMORIS study. Eur J Cancer. 2011;47:2033–41. 21486691 10.1016/j.ejca.2011.03.010

[49] Kunutsor SK, Laukkanen JA. Gamma-glutamyltransferase and risk of prostate cancer: findings from the KIHD prospective cohort study. Int J Cancer. 2017;140:818–24. 27861848 10.1002/ijc.30511

[50] Koenig G, Seneff S. Gamma-glutamyltransferase: a predictive biomarker of cellular antioxidant inadequacy and disease risk. Dis Markers. 2015;2015:818570. 26543300 10.1155/2015/818570 PMC4620378

[51] Almanza-Aguilera E, Martínez-Huélamo M, López-Hernández Y, Guiñón-Fort D, Guadall A, Cruz M, et al. Prediagnostic plasma nutrimetabolomics and prostate cancer risk: a nested case–control analysis within the EPIC study. Cancers (Basel). 2024;16:4116. 39682302 10.3390/cancers16234116 PMC11639937

